# High-Resolution LC–MS Characterization of *Ramaria flavobrunnescens,* a Coral Mushroom Toxic to Livestock, Reveals Fungal, Bacterial, and Eucalyptus Tree Metabolites

**DOI:** 10.3390/toxins18010053

**Published:** 2026-01-20

**Authors:** Megan J. Kelman, Justin B. Renaud, Joey B. Tanney, Mizael Machado, Mark W. Sumarah

**Affiliations:** 1 London Research and Development Centre, Agriculture and Agri-Food Canada, 1391 Sandford Street, London, ON N5V 4T3, Canada; megan.kelman@agr.gc.ca (M.J.K.); justin.renaud@agr.gc.ca (J.B.R.); 2 Pacific Forestry Centre, Canadian Forest Service, Natural Resources Canada, 506 Burnside Rd W, Victoria, BC V8Z 1M5, Canada; joey.tanney@nrcan-rncan.gc.ca; 3 Plataforma de Investigación en Salud Animal (PSA), Instituto Nacional de Investigación Agropecuaria (INIA), Estación Experimental Tacuarembó, Tacuarembó 45000, Uruguay; mmachado@inia.org.uy

**Keywords:** high-resolution mass spectrometry, *Ramaria* mushroom poisoning, mushroom endophytes, mycorrhizal symbiosis

## Abstract

*Ramaria flavobrunnescens* is an ectomycorrhizal coral mushroom that is often found growing in eucalyptus forests. The mushroom has been linked to accidental ingestion-associated livestock poisonings in South America, though the toxicological agent has not yet been described. Mushroom samples identified as *R. flavobrunnescens* were analyzed by liquid chromatography high-resolution mass spectrometry (LC–MS/MS) to determine the potential source of the toxicity, and to provide a metabolomic profile of the species. Previously reported *Ramaria* secondary metabolites were detected, including ramarins, ramariolides, pistillarin and arsenic-containing compounds. A number of bacterial species were isolated from *R. flavobrunnescens* that produced iron-chelating cyclic peptides, which were detected in the mushroom samples. Interestingly, we detected a series of eucalyptus tree secondary metabolites in abundance from *R. flavobrunnescens* fruiting bodies, some of which have reported toxicities and bioactivities. To our knowledge, this is the first report of eucalyptus secondary metabolites in a mushroom. The diversity of secondary metabolites identified in the mushroom extracts provides insight into the potential complex ecological interactions between *R. flavobrunnescens*, its associated microbiota, and its mycorrhizal interaction with eucalyptus trees.

## 1. Introduction

Mushrooms host a broad array of diverse biochemical capabilities due, in part, to occupying distinct niches globally, making them a rich source of bioactive natural products. While approximately 2000 mushroom species are classified as edible, it is estimated that approximately 643 mushroom species are known to be poisonous or capable of producing toxins [1,2]. The most commonly encountered mushroom toxicity comes from amatoxins, which are cyclic peptides predominantly produced across three genera: *Amanita*, *Lepiota*, and *Galerina* [3]. Beyond direct toxicity, mushrooms also play multifaceted ecological roles, with some ectomycorrhizal fungi characterized as facultative saprotrophs, and also harboring diverse fungal and bacterial communities associated with their mycelial mats and fruiting bodies [4,5].

**Figure 1:**
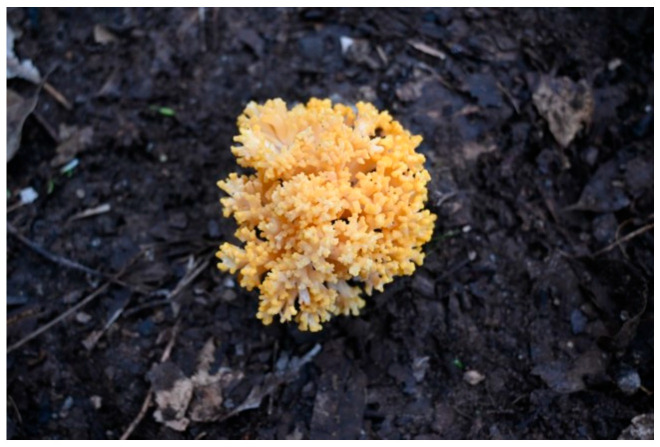
*Ramaria flavobrunnescens* fruiting body growing near a silvopastoral system in Uruguay.

*Ramaria flavobrunnescens* (also referred to as *R. flavo-brunnescens*) is a coral mushroom (Figure 1), named for its coral-like, dichotomously branching sexual fruiting bodies (basidiomata), and it belongs to the family *Gomphaceae*. The species apparently forms an ectomycorrhizal relationship with conifers and eucalyptus trees, and has a widespread distribution throughout Uruguay, Brazil, Argentina, Australia, and the southern United States. It is reported as the causative agent of accidental mushroom poisoning in eucalyptus forest-grazing livestock in temperate areas of South America, where it causes substantial economic impacts to farmers. Numerous spontaneous outbreaks have been recorded since the late 1950s in Uruguay, Argentina, and Southern Brazil, where silvopastoral agricultural systems are common practice, with outbreaks in some years resulting in over 96% herd fatality [6,7]. After ingestion, livestock experience acute gastrointestinal and neurological symptoms, including a characteristic loss of hair from the tail; loosening of the horn and hoof; ulcerative and gangrenous lesions on the skin, tongue, and esophagus; lameness; anorexia; and death in severe cases [8]. The causative agent(s) responsible for this toxicity has not been determined, likely due to the instability of the suspected toxins in the mushrooms after harvest. Previous studies that attempted to characterize the toxicological agents determined that the mushroom loses toxicity after being harvested or frozen, and is completely nontoxic after cooking or desiccation [9,10]. Due to the suspected instability of the compounds, it is believed that the causative agent is a thermolabile volatile alkaloid.

The symptoms and pathogenesis in cattle after controlled ingestion of fresh material were summarized in a review by Scheid et al. (2022) [8]. Symptoms appeared anywhere between three to eight days after ingestion, and pathology was suspected to be correlated to exposure to either trichothecenes, ergoalkaloids, or an unknown agent involved in the disruption of sulfur metabolism [11,12,13]. Chemical analysis is further complicated by the apparent obligate ectomycorrhizal dependence of *R. flavobrunnescens* on eucalyptus. We are unable to find reports of a method to isolate this fungus in pure culture. There are also abiotic factors that could potentially influence the toxicity [6]. Other *Ramaria* species have been classified as mildly poisonous and have been implicated in accidental poisonings. For example, if ingested in the fall months, *R. formosa* causes gastrointestinal issues due to an uncharacterized toxicological agent [14].

Modern analytical applications such as liquid chromatography tandem mass spectrometry (LC–MS/MS) can be used for both targeted and untargeted profiling, to either screen for known toxin classes, or for compound discovery. The best characterized species of *Ramaria* using LC–MS are the edible species, including *R. botrytis* and *R. flava* [15]. Therefore, the bulk of the *Ramaria* metabolomic profiling has centered around their nutritional composition, trace element accumulation, and their role in traditional medicine through profiling of the bioactive metabolites from the edible species. Several bioactive compounds, including fatty acids, phenolics, carotenoids, and antioxidants, have been previously characterized from edible *Ramaria* species by LC–MS [15,16,17]. Arsenic speciation was conducted on several species of *Ramaria* (*R. subbotrytis*, *R. largentii,* and *R. pallida*) using inductively coupled plasma mass spectrometry (ICP-MS) [18]. Other known *Ramaria* secondary metabolites include the ramarin sesquiterpenes from *R. formosa*, and ramariolides A–D from *R. cystidiophora* [19,20].

Despite the nutritional significance of some species of *Ramaria*, a more systematic examination of secondary metabolite production, especially for *R. flavobrunnescens*, has not been reported. In this study, we report the analysis of fruiting bodies of *R. flavobrunnescens* that were implicated in cattle toxicosis from Uruguay using high-resolution LC–MS to determine its secondary metabolite profile, and to determine any potentially toxic compounds or compound classes. The mushroom-associated bacteria and fungi were studied to assess their approximate contributions to the toxicity observed in outbreaks.

## 2. Results and Discussion

### 2.1. Determination of Secondary Metabolites from R. flavobrunnescens Extracts

From the initial mushroom pieces from Tacuarembó, Uruguay, material extracted with 79:20:1 acetonitrile: water: acetic acid resulted in the highest diversity of secondary metabolites (Supplementary Table S4). However, ethyl acetate was the best extraction method for nonpolar *Ramaria* secondary metabolites. Extraction of the lyophilized material yielded the highest number of detected peaks overall (*n* = 4884), compared to analysis of fresh weight material (*n* = 1539) (Supplementary Table S6). Unfortunately, many of the putatively identified compounds in the extracts could not be fully elucidated or quantified due to the lack of commercial standards available.

#### 2.1.1. Known Ramaria Secondary Metabolites

**Figure 2:**
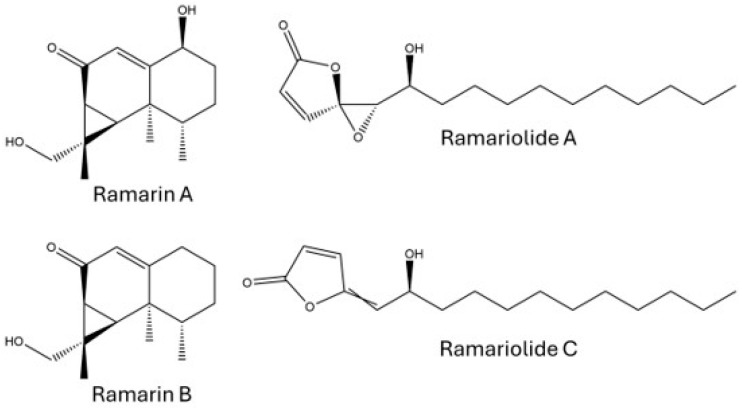
Known *Ramaria* secondary metabolites detected in mushroom extracts.

Extracts were first qualitatively investigated for any known secondary metabolites from the *Ramaria* genus. *R. flavobrunnescens* produced several compounds in the fresh weight ethyl acetate extracts that were previously reported from other *Ramaria* species, including the aristolane sesquiterpenes ramarin A/nambinone A (not distinguishable by MS/MS), ramarin B, axinysone A, and ramariolides (Figure 2). Of these, ramariolides A–D were detected in the highest abundances by peak area, with ramariolide A/B being the most abundant (Supplementary Figure S1). The ramarins were detected in low to modest amounts by peak area in the extracts. Kim et al. (2015) previously characterized the ramarin sesquiterpenes from the edible coral mushroom *R. formosa*, and reported minor inhibitory activity towards human neutrophil elastase [19]. Nambinones have also been described in *Neonothopanus nambi* (a bioluminescent mushroom), and were shown to have in vitro cytotoxicity towards a number of cancer cell lines [21]. Though their mammalian toxicity remains unclear, other aristolane-type sesquiterpenes and sesquiterpene lactones have reported cytotoxicity towards P-388 mouse leukemia cells, antimicrobial activity against *Staphylococcus aureus*, and can inhibit melanin synthesis [22,23,24]. The butanolide ramariolides, which were previously described in *R. cystidiophora*, showed antimycobacterial activity against *Mycobacterium smegmatis* and *M. tuberculosis* in cytotoxicity assays [20].

#### 2.1.2. Arsenic-Containing Compounds

**Figure 3:**
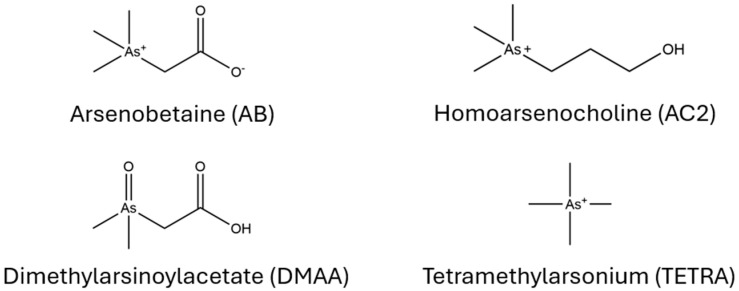
Arsenic-containing compounds detected in *Ramaria flavobrunnescens*.

Beyond the known secondary metabolites reported from *Ramaria*, the search was also expanded to include compounds reported across other mushroom genera. Several early-eluting compounds (~0.6 min) were observed in moderate abundance by peak area from samples extracted using the alkaloid extraction method—these were later identified as arsenic-containing compounds. Arsenobetaine (AB) (Figure 3) was identified as the major nontoxic arsenic-containing compound in *R. flavobrunnescens* based on published MS/MS fragmentation data [25,26]. We also putatively identified tetramethylarsonium (TETRA), dimethylarsinoylacetate (DMAA), and trace amounts of homoarsenocholine (AC2).

Arsenobetaine (AB) is known to be the major organic arsenical compound in most mushrooms, including other species of *Ramaria* [18,27,28]. Arsenobetaine has been hypothesized to act as an osmolyte to maintain structure of fruiting bodies, which potentially accumulates AB or arsenic-containing compounds from the environment [29]. Organic arsenic compounds are much less toxic than their inorganic forms, arsenate [As(V)] and arsenite [As(III)]. Arsenate has been shown to compete for phosphates, and can inhibit ATP production in cells [30]. Arsenite can reversibly bind the thiol moieties of reduced cysteine residues of enzymes [31]. It has been suggested that the microbial community and substrate may influence arsenic-speciation in mushrooms [29]. Some mushrooms, such as the mycorrhizal *Sarcosphaera coronaria* and *Cyanoboletus pulverulentus,* can accumulate arsenic in their fruiting bodies as methylarsonic acid (MA) or dimethylarsonic acid (DMA) [32]. Interestingly, *S. coronaria* also has an unknown toxicological origin with sporadic toxicity. While inorganic arsenic remains acutely more toxic than organic arsenic or arsenicals, the presence of iron-chelating siderophores may increase the bioavailability of inorganic arsenic [33,34]. Arsenic-containing compounds have been shown to change their speciation after cooking or after ingestion [35,36,37]. Though arsenic poisoning may potentially mimic a few of the symptoms, it is unlikely to be the causative agent of *R. flavobrunnescens*-associated poisoning.

#### 2.1.3. Iron-Chelating Siderophores and Cyclic Nonapeptides

**Figure 4:**
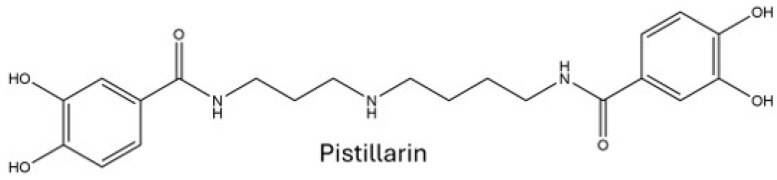
The structure of the iron-chelating siderophore pistillarin.

Beyond known mushroom toxins and secondary metabolites, all extracts were investigated for a broad range of microbial natural products. The known iron-chelating siderophore pistillarin (Figure 4) was detected in minor amounts from the 79:20:1 acetonitrile: water: acetic acid mushroom extracts using the GNPS MS/MS library search database for compound dereplication.

Pistillarin has previously been described from *Ramaria botrytis* [38]. Both pistillarin and pistillarin B (not detected in *R. flavobrunnescens* in this study) showed antilipid peroxidation activity [38]. Mushrooms have previously been reported to have antilipid peroxidation activity if they are grown in particularly UV-exposed or polluted areas [39]. Sakemi et al. (2022) suggested that *Ramaria* species, which can have ectomycorrhizal relationships with plants, may have a role in plant protection by secreting pistillarin when plants are under oxidative stress [38]. It is thought to inhibit lipid peroxidation by chelating iron or other metals involved in peroxidation. Pistillarin has also been reported from filamentous fungi, such as *Penicillium bilaiae*, though its role is predominantly iron acquisition in iron-depleted environments [40].

**Figure 5:**
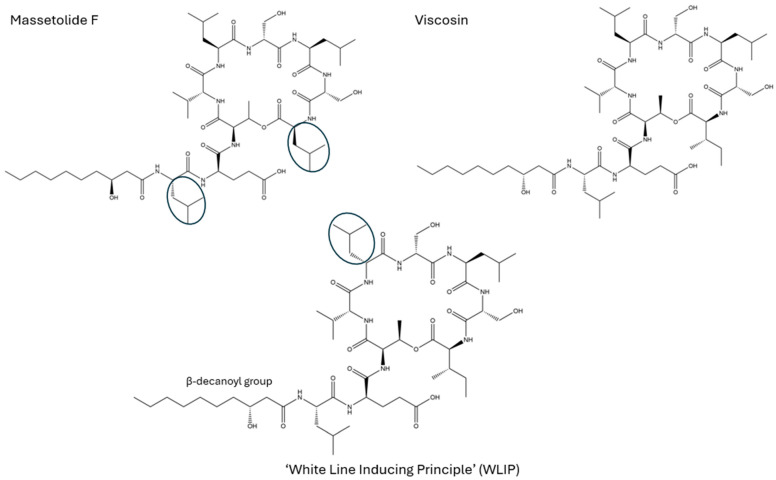
Structures of cyclic nonapeptides putatively detected in *R. flavobrunnescens* extracts with β-decanoyl group, and diversity of stereochemistry between structures. Circled areas highlight the amino acid stereochemistry differences between the compounds.

Interestingly, freeze-dried mushroom extracts also revealed large peptide-containing clusters between 1100 and 1200 Da, which also were shown to have iron adducts in their mass spectra (Δ 53.9396 Da). Peptide sequencing using MS/MS revealed that these compounds belonged to the WLIP/viscosin/massetolide family of cyclic nonapeptides (Figure 5) [41]. They exist in both cyclic and straight-chain forms and contain a characteristic β-decanoyl group bound to an *N*-terminal leucine residue. It is unclear which class the compounds belong to as their differentiation is either a leucine/isoleucine, or different L/D confirmation, which cannot be differentiated without derivatization.

The WLIP/massetolide/viscosin compounds have not previously been reported from mushrooms. They are commonly associated with *Pseudomonas* species in the *fluorescens* species complex, which have been detected in a wide range of environmental substrates, including mushrooms [42]. The compounds are all bioactive, though their biological activities differ. All three compounds have similar modular nonribosomal peptide synthase (NRPS) biosynthetic gene clusters [43]. WLIP (White Line Inducing Principle) is antifungal, inhibits the growth of Gram-positive bacteria, and has been shown to affect membrane permeability [41,44]. Massetolides and viscosin have been shown to be active against mycobacteria and have roles in biofilm formation and rapid host colonization [43,45,46]. Viscosin-producing *P. fluorescens* was shown to influence soil microbiota in wheat cultivars, suggesting a possible plant–bacteria relationship. Cucumber plants have been shown to absorb and mobilize iron through their roots when iron-chelated siderophores were supplied hydroponically [47]. Iron cycling has been shown to have a crucial role in white rot fungi in order to degrade the lignin and cellulose in woody substrates via the Fenton reaction [48,49]. This suggests a potential means of ectomycorrhizal iron cycling between the tree and the mushroom, or between the mushroom and the bacteria.

Due to the overall relative abundance in the samples (by peak area) and potential microbial origins of the identified WLIP/massetolide/viscosin compounds, mushroom pieces were surface-sterilized and plated to determine the potential source. Two bacterial species were isolated from surface-sterilized mushroom segments. The 16S bacterial sequencing confirmed that they were *Pseudomonas fluorescens* and the known mushroom pathogen *Ewingella americana*. The liquid culture extracts from *P. fluorescens* and *E. americana* isolates were then analyzed by LC–MS/MS analysis to determine whether any abundant compounds were also detected in the *R. flavobrunnescens* extracts.

*Ewingella americana* is a rare Gram-negative bacteria from the Enterobacteriaceae family that was described in 1983 [50]. It is a known mycopathogen of the edible mushrooms *Agaricus bisporus*, *Lentinula edodes*, *Pleurotus ostreatus*, and *Flammulina velutipes*, causing internal stipe necrosis and brown blotch disease [51,52,53]. The species has a global distribution and wide host range, also isolated from decomposing fungal biomass, soil, mollusks, nutria, vacuum packaged beef, and humans [54,55,56,57,58,59]. The species expresses chitinases, has demonstrated phenol biodegradation capability, and is resistant to several antibiotics [54,60,61].

Under the conditions tested, the *Ewingella americana* extract did not contain compounds that were also detected in the mushroom fruiting bodies. However, the *P. fluorescens* extract was shown to produce the cyclized version of the WLIP/massetolide/viscosin family, as well as the iron adduct versions, indicating that these compounds were not biosynthesized by the fungus.

A number of microbial endophytes and saprotrophs have been characterized in edible mushrooms and are predominantly reported as *Pseudomonas* species. Some saprophytic bacterial species from the *Pseudomonas reactans* species group have been isolated from the cultivated mushroom *Agaricus bisporus* (white button mushroom) and shown to produce WLIP lipodepsipeptides [62]. Berendsen et al. (2012) screened 160 strains of *Pseudomonas* species isolated from mushrooms, and determined that the mushroom pathogen *Lecanicillium fungicola* (causal agent of dry bubble disease) was extremely sensitive to siderophore production and competition for iron [63]. The production of WLIP has been detected in the *Pseudomonas reactans* species group, though cultured strains appear to lose virulence and the ability to produce WLIP [64]. This suggests that WLIP is a potential virulence factor and that its production is linked to the mushroom–bacterial interaction.

#### 2.1.4. Formylated Phloroglucinol Compounds (FPCs) from Eucalyptus

**Figure 6:**
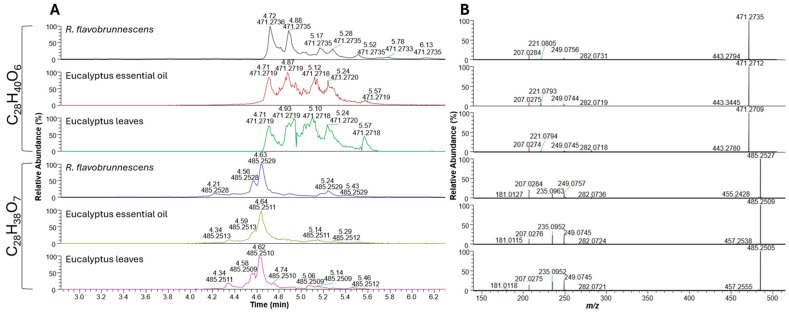
(**A**) Extracted ion chromatograms highlighting major eucalyptus compounds detected in *R. flavobrunnescens* extracts compared to extracts of eucalyptus essential oil and fresh eucalyptus leaves. (**B**) Associated MS/MS fragmentation of *m*/*z* 471.2757 and 485.2527 [M-H]^−^ at most intense MS/MS peaks in the mushroom, essential oil, and eucalyptus leaf extracts.

After identifying known secondary metabolites from fungal and bacterial origin, the mushroom extracts were qualitatively analyzed using nontargeted analysis (NTA). Two major unknown compounds (C_28_H_40_O_6_ and C_28_H_38_O_7_) were initially detected in high abundance (by peak area) from ethyl acetate extracts of *R. flavobrunnescens* in negative ionization mode (Figure 6A). Using the GNPS dereplicator module, these compounds were putatively identified to be eucalyptin A and eucalyptone, which are a class of formylated phloroglucinols (FPCs) known from eucalyptus trees. FPCs are a structurally diverse class of bioactive secondary metabolites produced by members of the large eucalyptus family [65,66]. Based on published MS/MS transitions of known FPCs, the two abundant compounds by peak area were putatively confirmed to be in the same compound class but could not be elucidated to an individual compound due to the diversity of isobaric FPCs. For additional confirmation, mushroom extracts were then compared to extracted raw eucalyptus leaves and extracted eucalyptus essential oil (Figure 6A,B). Detected compounds were compared to commercially available analytical standards to determine the most abundant FPC compounds in the *Ramaria* extracts. A C8 column was used for better resolution of structurally related FPCs.

Macrocarpals A, C, and N were the only FPC compounds that could be positively identified in the *R. flavobrunnescens* extracts based on both the availability and quality of commercial standards (Supplementary Figures S3–S5, Supplementary Table S3). Euglobals Ia1 and Ia2 had similar retention times to the putatively identified euglobals in the mushroom extracts, but had differing fragmentation patterns; thus, different unidentified euglobals are likely present (Supplementary Figure S3). Macrocarpals D and E were not detected in the *Ramaria* ethyl acetate extracts (Supplementary Figure S5). Macrocarpal A is a major FPC in eucalyptus leaves and was also found in the highest concentration in the *R. flavobrunnescens* sample (60.7 µg/kg) [65,66,67,68]. Lower amounts of macrocarpal C (21.3 µg/kg) and N (24.5 µg/kg) were determined in the same sample.

**Figure 7:**
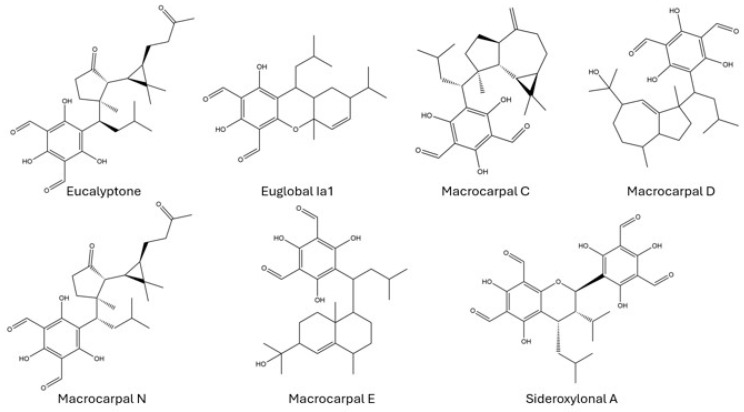
Putative formylated phloroglucinol compounds (FPCs) in ethyl acetate mushroom extracts with commercial standards available. Euglobal Ia1, macrocarpal D, and macrocarpal E were not detected in ethyl acetate extracts.

Eucalyptone and macrocarpal N (Figure 7) are sesquiterpene macrocarpals with reported antibacterial activity [69,70]. Other macrocarpals (including eucalyptone) have been shown to inhibit glucosyltransferase activity, which is linked to biofilm formation or peptidoglycan synthesis in microbes [71,72,73,74]. Sideroxylonals from eucalyptus species are the primary antiherbivory compounds involved in directly affecting foraging behaviour through their bitter taste [75]. Eucalyptus extracts have antimicrobial, antiviral, and antiinsectan activity [76,77,78,79,80,81]. Eucalyptus essential oils and extracts have reported toxicity in humans in high doses [82,83,84]. This is predominantly attributed to 1,8-cineole (eucalyptol) and cyanogenic glucosides. Symptoms of eucalyptus poisoning includes GI issues (abdominal pain, vomiting) as well as central nervous system effects (disorientation, drowsiness, poor muscle control, and tremors), convulsions, hypoventilation, rapid heart rate, drooling or frothing, and issues with temperature regulation (resulting in hypothermia or hyperthermia) [85,86,87,88]. Eucalyptol is a volatile terpene, and is susceptible to light and temperature degradation [89]. Neither eucalyptol nor cyanogenic glucosides could be detected in the initial extracts, but the high relative abundance of other eucalyptus compounds may imply their presence and warrants more targeted analysis of fresh material to confirm.

Due to the peak area abundance of the FPCs in the *R. flavobrunnescens* extract, it was suspected that more related compounds were present. To determine whether any additional structurally related FPC compounds were in the *R. flavobrunnescens* extracts, the known diagnostic product ions for the structurally conserved regions of macrocarpals (*m*/*z* 207.0274) and sideroxylonals (*m*/*z* 249.0747, 181.0119) were used for product ion filtering (Supplementary Figure S1) to find additional related compounds [65]. Using product ion filtering, 40 potentially distinct structurally related compounds were identified with shared fragmentation pathways for macrocarpals and sideroxylonals. Compounds were shown to primarily elute between 4 and 6 min, with most formulae representing between 3 and 5 different isobaric peaks (Supplementary Table S2). Of the 40 molecular formulae, 27 were unable to be putatively identified from known FPCs. Some 38 molecular formulae were found in eucalyptus leaf extracts, and 19 were found in the eucalyptus oil extract. Eight molecular formulae were solely found in the ethyl acetate mushroom extract.

Profiling of known FPCs by LC–MS has been previously accomplished by Okba et al. (2017, 2021) and Dos Santos et al. (2019) in leaves, flowers, and flower buds across several eucalyptus species [65,68,90]. Due to the nonpolar nature and structural diversity of the FPCs, it is an analytical challenge to fully elucidate and quantify all related compounds. Semi-quantification of structurally related isobaric compounds using a surrogate standard has been used previously in other plant matrices. Similarly, Roridin A was used for the estimation of total macrocyclic trichothecenes in *Baccharis coridifolia* as ‘roridin A equivalents’ [91]. Dos Santos et al. (2019) used commercial standards of macrocarpal A and sideroxylonal A for the estimation of total macrocarpals and sideroxylonals, respectively, in eucalyptus matrices [65]. Following previous works on semi-quantification using surrogate compounds by Santos et al. (2019) [65] and Machado et al. (2023) [91], the quantities of the other major FPCs in the *Ramaria* extracts are instead estimated here as “macrocarpal A equivalents”. For the 40 related FPCs detected by product ion filtering, it can be estimated that the original *R. flavobrunnescens* extract had a total of 5.36 µg/g macrocarpal A equivalents. The molecular formulae with the highest concentrations were C_26_H_28_O_10_ (*m*/*z* 499.1609 [M-H]^−^; 3.17 µg/g), C_28_H_40_O_6_ (*m*/*z* 471.2736 [M-H]^−^; 0.483 µg/g), C_28_H_38_O_5_ (*m*/*z* 453.2633 [M-H]^−^; 0.389 µg/g), C_28_H_38_O_7_ (*m*/*z* 485.2535 [M-H]^−^; 0.358 µg/g), and C_33_H_36_O_11_ (*m*/*z* 635.2842 [M-H]^−^; 0.186 µg/g).

This may represent one of the first reports of plant-derived compounds bioaccumulating in mushroom fruiting bodies. While eucalyptus compounds have not yet been reported to bioaccumulate in mushrooms, other edible mushroom species, including *Pleurotus ostreatus* and *Favolus tenuiculus*, have been reported to biotransform 1,8-cineole (eucalyptol) from eucalyptus waste into two new related oxygenated derivative compounds [92]. Monoterpenes and sesquiterpenes are naturally abundant in soil, woody material, and decomposing leaf material, with some studies supporting that terpenes affect soil nutrient cycling and decomposition. This suggests the possibility that *R. flavobrunnescens* and other ectomycorrhizal mushrooms may have the potential to adapt to potentially toxic variants of terpenes (such as eucalyptol) [93,94]. While some plant-associated fungi can detoxify host defense compounds [95,96,97], bioaccumulating them in fruiting bodies may indicate a broader capacity to sequester, transform, or tolerate specialized plant metabolites, with the additional advantage of providing antifeedant properties that may reduce grazing pressure on fruiting bodies.

Alternatively, some endophytic fungi can produce the same or analogous biologically active secondary metabolites as their plant hosts, including those with antifungal properties, such as camptothecin [98,99,100]. This may be driven by horizontal gene transfer or convergent evolution of biosynthetic gene clusters. Kusari et al. (2011) hypothesized that fungal endophytes intrinsically possess resistance to antagonistic host secondary metabolites, such as camptothecin, and may later acquire additional co-evolutionary resistance traits through prolonged host–endophyte interaction [101]. The fungus *Inonotus obliquus*, known as the chaga mushroom and widely used for therapeutic and medicinal purposes, is a pathogen of birch (*Betula*) trees. Both *I. obliquus* and its host tree produce the triterpenoids betulin and betulinic acid, with evidence suggesting that betulin biosynthesis evolved independently in the fungus through long-term co-evolution with its host [102]. Another decay fungus, *Fulvifomes xylocarpicola*, produces fruiting bodies that contain the same limonoid triterpenoids as its host, although the origin of these secondary metabolites remains unknown [103]. Ecologically, plant–fungus associations may influence the secondary metabolite profiles of fungal fruiting bodies, with implications for fungal fitness and interactions with other organisms. Future elucidation of the origin of FPCs in the *Ramaria* mushrooms may include comparative genomic approaches to identify putative terpene, polyketide, or hybrid biosynthetic gene clusters, complemented by transcriptomic analyses to assess the expression of fungal terpene synthases from mushrooms. In addition, stable-isotope labeling could determine whether the metabolites incorporate fungal-derived carbon (supporting endogenous biosynthesis) or host-derived carbon (indicating uptake or transformation of plant compounds).

The estimation of total macrocarpal A equivalents is likely a low-end approximation of the actual FPCs present in fresh fruiting body material, as their fate and persistence in the plant–mushroom–microbiota niche is unknown. It is also unknown whether FPCs can be further biotransformed by associated microorganisms occupying the same niche. The estimated amount is also unlikely to be solely responsible for the extent of livestock poisoning reported in South American pastures. The suspect toxin(s) is known to be unstable from previous analyses, so the active agent(s) may not have been present in the original sample materials at the time of analysis. Although the mushroom was sampled from a pasture with previous livestock poisonings, it was unclear if this mushroom specimen was directly associated with any toxicological effects. Future studies will need to ensure additional sampling considerations to better preserve the volatiles and potentially unstable compounds.

## 3. Conclusions

Both targeted and nontargeted analysis using high-resolution LC–MS/MS were used for a comprehensive secondary metabolite profile from *R. flavobrunnescens*. Known *Ramaria* compounds were identified, including arsenic-containing compounds, and previously described sesquiterpenes. Iron-chelating nonapeptides were observed in mushroom fruiting body extracts and were determined to originate from bacterial isolates from *R. flavobrunnescens* material. Nontargeted analysis of the fruiting body material determined that the most abundant compounds were FPCs from eucalyptus. The unexpected relative abundance from both plant and microbial sources warrants further study into the dynamic plant–mushroom–microbial communities in eucalyptus understories. This will include comparative whole-genome sequencing for mining of candidate toxigenic clusters, and determination of the potential biosynthetic rationale behind FPC bioaccumulation in *R. flavobrunnescens* fruiting bodies. Despite the integrative analysis into *R. flavobrunnescens*, the toxic agent responsible for cattle deaths still cannot be unequivocally determined and warrants further analysis of toxicosis-linked material.

## 4. Materials and Methods

### 4.1. Ramaria flavobrunnescens Identification and Sample Collection

Twenty mushroom samples were collected from the base of *Eucalyptus camaldulensis* trees by the Department of Tacuarembó during May 2024 (autumn in the Southern Hemisphere) on a farm in Uruguay with a previous history of *Ramaria flavobrunnescens* poisoning in cattle. The samples were labeled 1–20 and stored at −80 °C. For shipment, they were kept on dry ice, and mushroom pieces were placed into 1.7 mL microcentrifuge tubes for LC–MS analysis.

### 4.2. Chemicals and Analytical Standards

Optima LC–MS-grade water, acetonitrile, methanol, and formic acid (Fisher Scientific, Hampton, NH, USA) were used for sample reconstitution, mobile phase, and buffer preparation. Ethyl acetate, hexane (ACS grade), acetic acid (ACS Plus), and ammonium bicarbonate were purchased from Fisher Scientific. Analytical standards of macrocarpals A, C, D, E, N, euglobal Ia1, 1a2, and eucalyptone were purchased from Biosynth (Staad, Switzerland). Sideroxylonal A was purchased through MedChemExpress (Monmouth Junction, NJ, USA).

### 4.3. Sample Extraction

Mushroom pieces from the first batch of samples (July 2024) were roughly divided into five groups and then split in half (*n* = 10 pieces of mushroom, ~1 g fresh weight) of approximately equal size. Half of the mushroom pieces were lyophilized (Labconco Freezone, MO, USA), and the other half of the pieces were extracted as raw segments. Various methods were tested to determine optimal extraction conditions for a comprehensive representation of secondary metabolites in the mushroom samples). Mushroom pieces were separately extracted (raw and lyophilized) in either (1) 79:20:1 acetonitrile: water: acetic acid (*v*/*v*/*v*), (2) hexane, (3) ethyl acetate, (4) methanol, or by (5) alkaloid extraction [104,105]. For extraction methods 1–4, raw and freeze-dried segments were added separately to 7 mL glass scintillation vials with 2 mL of specified solvent. The solutions were vortexed for 30 s and then sonicated at room temperature for 1 h. The supernatant from each vial was removed, added to clean 7 mL glass scintillation vials, and dried under nitrogen. Extracts were reconstituted in 1 mL acetonitrile, vortexed briefly, and filtered through 0.45 µm PTFE syringe filters (Fisher Scientific) into 2 mL amber HPLC vials (Agilent Technologies, Santa Clara, CA, USA) for analysis. The alkaloid extraction (5) was performed as per Di Mavungu et al. (2012), with some deviations [105]. Briefly, 10 mL portions of ethyl acetate phase were dried, and extracts were reconstituted in 400 µL of 20/40/40 methanol/acetonitrile/water.

### 4.4. LC–MS/MS Analysis

For LC–MS/MS screening of extraction methods 1–4, an Agilent 1290 Infinity HPLC was coupled to a Thermo Q-Exactive Orbitrap mass spectrometer (Thermo Fisher Scientific, Waltham, MA, USA). For compound separation, 5 µL of each sample were injected onto an Zorbax Eclipse Plus RRHD column (2.1 × 50 mm, 1.8 µm; Agilent) held at 35 °C at a flow rate of 0.3 mL/min. Separation was achieved using mobile phases composed of Optima-grade H_2_O with 0.1% formic acid (A) and Optima-grade ACN with 0.1% formic acid (B). Mobile phase B was held at 0% for 30 s before ramping to 100% over 3 min. B was held at 100% for 2.5 min before ramping back to 0% over 30 s. Data dependent acquisition (DDA) was used in both positive and negative ionization modes for each sample using the HESI source. The following settings were used for DDA acquisition by full MS: scan range, 150–1600 *m*/*z*; resolution, 70,000; automatic gain control (AGC), 1 × 10^6^; and max injection time (IT), 256 ms. The top 5 peaks from each full MS scan (by intensity) were selected for further analysis by MS/MS under a 1.2 *m*/*z* isolation window using the following settings: resolution, 17,500; AGC target, 1 × 10^5^; max IT, 62 ms; NCE, 35; threshold intensity, 1.2 × 10^5^; dynamic exclusion, 10 s. Analysis of eucalyptus standards was similarly investigated with different column chemistries to evaluate separation, including the Zorbax Eclipse Plus C8 RRHD (2.1 × 50 mm, 1.8 µm; Agilent) and an Accucore Phenyl-Hexyl (2.1 × 50 mm, 2.6 µm; Thermo Scientific). For the alkaloid extracts, chromatography conditions were adopted using Di Mavungu et al. (2012), using water/0.2 M ammonium bicarbonate pH 10/methanol (85/5/10 *v*/*v*/*v*, mobile phase A), and water/0.2 M ammonium bicarbonate pH 10/methanol (5/5/90 *v*/*v*/*v*, mobile phase B) [105]. All raw data files were analyzed using Thermo Xcalibur software using the Qual browser, Mzmine v. 4.1.0, and xcms and MetabolAnalyze packages in R v. 4.4.1 (r-project.org) using mzml converted files (Proteowizard) [106,107,108]. Peak lists were generated using R per Kelman et al. (2020) [109]. Compounds were identified using available analytical standards, previously compiled in-house databases, and comparison to published natural product spectral libraries in GNPS, Metabolomics of North America (MoNA), and MassBank. All detected abundant compounds were assigned a confidence ID (1–5) based on their comparison to analytical standards (1), spectral database matching (2), in-silico MS/MS, putative matches of known secondary metabolites or compound classes (3), or chemical formula (4).

#### Quantification and Semi-Quantification of Formylated Phloroglucinol Compounds

Due to the limited sample material available, only a single mushroom piece (*n* = 1, 235.3 mg fresh weight) could be used for quantification, so biological replicates could not be reported. Macrocarpals A, C, and N were quantified using commercial standards prepared neat in acetonitrile from 1 ng/mL to 10 µg/mL. Based on previous works, macrocarpal A was used as the surrogate compound in the semi-quantification of the total FPCs in the *R. flavobrunnescens* extract, as it was quantified in the highest amount [65,91]. Product ion filtering using the macrocarpal and sideroxylonal known product ions (*m*/*z* 207.0274, 249.0747 and 181.0119, [M-H]^−^) was used to detect the molecular formulae of the total FPCs in the *R. flavobrunnescens extract* [65]. The peak areas of each molecular formulae were integrated in the full MS, and semi-quantified as macrocarpal A equivalents. The total FPC for the extract is reported as a sum of the integrated peak areas as macrocarpal A equivalents.

### 4.5. Surface Sterilization and Isolation of Microorganisms from Mushroom Segments

Small pieces of the mushroom (~1 cm × 0.5 cm) were surface-sterilized by briefly swirling in 95% ethanol for 1 min and then washing with sterile distilled water. Pieces were aseptically plated onto Malt Extract Agar (MEA) plates (Millipore Sigma, Burlington, MA, USA) and incubated at 25 °C until growth was observed. Individual fungal and bacterial colonies were streaked onto new MEA agar plates to select for single colony isolates. For each isolate, DNA was extracted (DNeasy UltraClean Microbial Kit, Qiagen, Toronto, ON, Canada) and amplified by PCR using either 16S bacterial 27F/1492R primers or ITS1/ITS4 primers (10 µM stocks, 1.5 µL each, Supplementary Table S1) in 25 µL reaction mixtures using 20 µL SuperMix (Thermo Platinum Blue PCR SuperMix) and 2 µL DNA. Amplified PCR products were cleaned with ExoSAP-IT (Thermo Fisher, manufacturer’s instructions), and sent to Eurofins (Louisville, KY, USA) for Sanger sequencing analysis (Eurofins SimpleSeq pre-paid tubes).

### 4.6. Growth and Extraction of Extrolites from Single Colony Isolates

Single colonies of bacteria from each isolate were inoculated into 7 mL Trypticase Soy Broth (TSB, BD BBL, Franklin Lakes, NJ, USA) in 15 mL conical tubes and capped with sterile foam plugs. Cultures were incubated in a shaking incubator maintained at 30 °C at 200 rpm for 48 h. Cultures were extracted using 7 mL of the mycotoxin mix (79:20:1 acetonitrile: water: acetic acid) in a 50 mL conical tube. Briefly, samples were sonicated at 30 °C for 30 min and then shaken on an orbital shaker at 250 rpm for 15 min. Conical tubes were centrifuged at 3220× *g* at 4 °C for 10 min. 500 µL of the supernatant was diluted with 500 µL of 50:50 acetonitrile: water, and syringe filtered using 0.45 µM PTFE filters into 2 mL amber HPLC vials. Samples were analyzed by LC–MS/MS as described above, but also with an inclusion list for the known WLIP/massetolide/viscosin compounds. Raw files were also converted into mzml file format (MS Convert) and analyzed using the GNPS library search. For fungal cultures, six agar plugs were excised from single colony isolates (MEA) using a 0.5 cm diameter cork borer into a 7 mL glass scintillation vial. Agar plugs were extracted with 2 mL ethyl acetate by sonication at 30 °C for 60 min in a sonicating water bath. The supernatant was removed and evaporated to dryness under a gentle stream of nitrogen, reconstituted in 1 mL acetonitrile, and syringe-filtered using 0.45 µM PTFE filters into 2 mL amber HPLC vials for LC–MS analysis.

### 4.7. Extraction of Fresh Eucalyptus Leaves and Essential Oil

The *Eucalyptus* ‘Glacier’ cultivar was purchased from a commercial greenhouse in Thorndale, Ontario. Briefly, 1.12 g of fresh weight eucalyptus leaves were harvested, manually torn, and ground using a mortar and pestle in 5 mL ethyl acetate. The organic extract was filtered through P1 filter paper with anhydrous sodium sulfate into a round-bottomed flask and evaporated to dryness on a rotary evaporator. The extract was reconstituted in approximately 2 mL ethyl acetate and evaporated to dryness using a centrivap vacuum concentrator (Labconco). For LC–MS analysis, the dried extract was reconstituted in 1 mL acetonitrile, diluted 100× in acetonitrile, and syringe-filtered by 0.45 µM PTFE into 2 mL amber HPLC vials.

Eucalyptus essential oil extract from *Eucalyptus globulus* was purchased locally. For the essential oil, 1 mL was extracted with 5 mL ethyl acetate. The organic portion was evaporated to dryness using a centrivap concentrator and reconstituted in 1 mL acetonitrile with sonication at 30 °C for 10 min. The extract was diluted 10× in acetonitrile, and syringe-filtered using 0.45 µM PTFE into 2 mL amber HPLC vials for LC–MS analysis.

## Data Availability

The data presented are available in the article and Supplementary Materials.

## Supplementary Materials

The following supporting information can be downloaded at: https://www.mdpi.com/article/10.3390/toxins18010053/s1. Table S1: Bacterial and fungal primers used for Sanger sequencing of isolated microorganisms from *Ramaria flavobrunnescens*; Table S2: Structurally related formylated phloroglucinol compounds (FPCs) detected by product ion filtering of *m*/*z* 207.0274 [M-H]^−^ from the *Ramaria* ethyl acetate extract. Compounds were then screened for their presence in eucalyptus leaf and essential oil extracts; Table S3: Retention time and high-resolution MS/MS fragmentation of commercially available eucalyptus standards; Table S4: Knowns and putative identifications; Table S5: Unknown compounds; Table S6: Peak tables showing differences between lyophilized and raw mushroom material; Figure S1: (A) Extracted ion chromatograms of the putatively identified ramariolides A–D in ethyl acetate extracts in negative ionization mode, highlighting relative abundances between them. (B) MS/MS of the putative ramariolides A/B, D, and C [M-H]^−^ from *R. flavobrunnescens* extracts; Figure S2: Product ion filtering in negative ionization mode of diagnostic fragment *m*/*z* 207.0274 for the *Ramaria* ethyl acetate extract, extracted eucalyptus leaves, and eucalyptus oil. The peaks and relative abundances detected highlight the diversity of the formylated phosphoglucinol compounds (FPCs) between the matrices; Figure S3: (A) Extracted ion chromatograms of 385.2029 [M-H]^−^ and (B) MS/MS for the *Ramaria* extract, euglobal Ia1, and euglobal Ia2; Figure S4: (A) Extracted ion chromatogram of 485.2568 [M-H]^−^ and (B) MS/MS fragmentation for the *Ramaria* extract, eucalyptone, and macrocarpal N; Figure S5: (A) Extracted ion chromatogram of 471.2772 [M-H]^−^ and (B) MS/MS fragmentation of the *Ramaria* ethyl acetate extract and macrocarpals A, D, and E; Figure S6: (A) Extracted ion chromatogram of 499.1609 [M-H]^−^ and (B) MS/MS fragmentation of the *R. flavobrunnescens* extract and sideroxylonal A; Figure S7: (A) Extracted ion chromatogram of 453.2646 [M-H]^−^ and (B) MS/MS fragmentation of the *R. flavobrunnescens* extract and macrocarpal C.

## Abbreviations

The following abbreviations are used in this manuscript:

GNPS	Global Natural Products Social Molecular Networking
HRMS	High-resolution mass spectrometry
LC–MS/MS	Liquid chromatography tandem mass spectrometry
TSB	Trypticase soy broth
WLIP	White line inducing principle
FPC	Formylated phloroglucinol compound
