# Supplementary material for: High-Resolution LC–MS Characterization of Ramaria flavobrunnescens, a Coral Mushroom Toxic to Livestock, Reveals Fungal, Bacterial, and Eucalyptus Tree Metabolites
Source: Toxins (Basel). 2026 Jan 20;18(1):53. doi: 10.3390/toxins18010053 (PMC12845983; doi:10.3390/toxins18010053)
Supplement: Supplementary file 1 [file toxins-18-00053-s001.zip › Supplementary information.pdf]

Supplementary information

Table S1. Bacterial and fungal primers used for Sanger sequencing of isolated microorganisms from *Ramaria flavo-brunnescens*

Primer	Sequence
27F	AGAGTTTGATCMTGGCTCAG
1492R	TACGGYTACCTTGTTACGACTT
ITS1	TCCGTAGGTGAACCTGCGG
ITS4	TCCTCCGCTTATTGATATGC

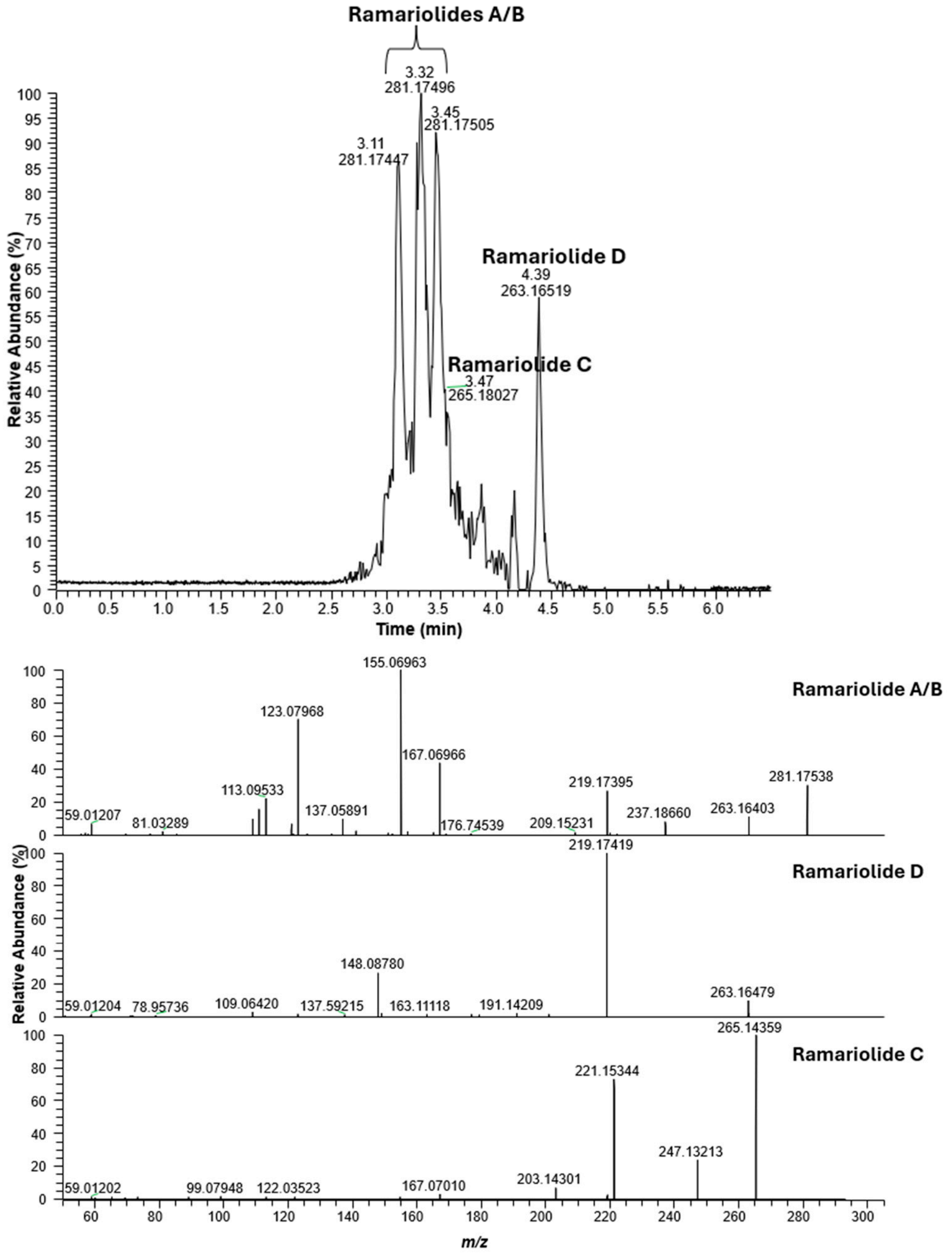

Figure S1- A) Extracted ion chromatograms of the putatively identified ramariolides A-D in ethyl acetate extracts in negative ionization mode, highlighting relative abundances between them **B)** MS/MS of the putative ramariolides A/B, D and C $[M-H]^-$ from *R. flavo-brunnescens* extracts

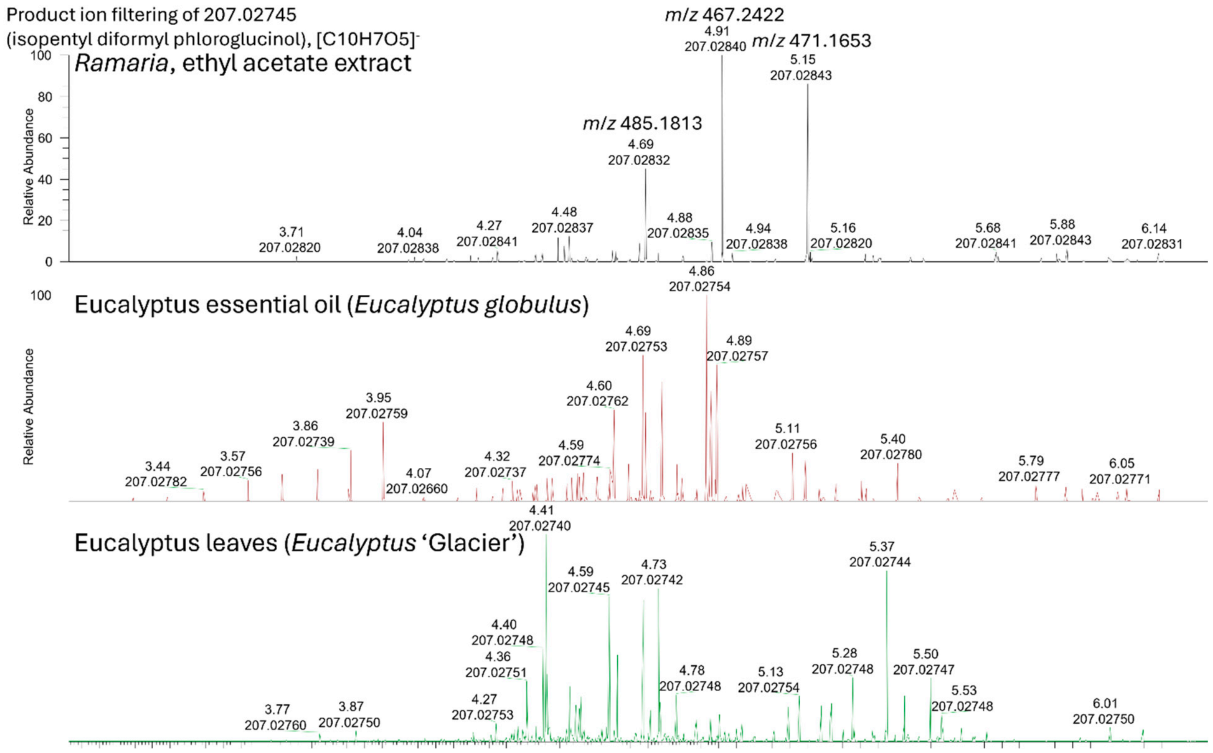

Figure S2- Product ion filtering in negative ionization mode of diagnostic fragment *m/z* 207.0274 for the *Ramaria* ethyl acetate extract, extracted eucalyptus leaves, and eucalyptus oil. The peaks and relative abundances detected highlight the diversity of the formylated phosphoglucinol compounds (FPCs) between the matrices.

Table S2- Structurally related formylated phloroglucinol compounds (FPCs) detected by product ion filtering of *m/z* 207.0274 [M-H]- from the *Ramaria* ethyl acetate extract. Compounds were then screened for their presence in eucalyptus leaf and essential oil extracts

FPC #	*m/z*	Molecular formula	t_R (min)	Product ion(s)	Putative ID(s)	Macrocarpal A equivalent (µg/g)	Detected in
1	357.1335	$C_{20}H_{22}O_6$	4.01	207.0274	Eucalypcamal F	<LOQ	Mushroom, leaves
2	379.1750	$C_{20}H_{28}O_7$	4.59	207.0274, 249.0747, 282.0734		<LOQ	Mushroom, leaves
3	381.1698	$C_{23}H_{36}O_5$	4.79, 5.19, 5.43, 4.21	207.0274, 261.2033		<LOQ	Mushroom, leaves, oil
4	383.1852	$C_{23}H_{28}O_5$	4.97, 4.87	207.0274, 249.0747		0.0474	Mushroom, leaves, oil

FPC #	*m/z*	Molecular formula	t_R (min)	Product ion(s)	Putative ID(s)	Macrocarpal A equivalent (μg/g)	Detected in
5	385.2009	$C_{23}H_{30}O_5$	5.37, 5.19, 5.88, 6.16, 5.04, 4.75	207.0274, 249.0747	25 euglobals, or robustadial A/B	0.0630	Mushroom, leaves, oil
6	399.1799	$C_{23}H_{28}O_6$	4.21, 4.45, 4.88, 5.19, 5.47	207.0274, 249.0747	Eucalypcamal B	<LOQ	Mushroom, leaves, oil
7	401.1954	$C_{23}H_{30}O_6$	4.51, 4.65, 4.76, 4.92, 5.04, 5.17, 5.24, 5.42, 5.67, 6.03	249.0747, 207.0274, 181.0124	Eucalypglobulusal J	0.0204	Mushroom, leaves, oil
8	413.1591	$C_{23}H_{26}O_7$	5.20	249.0747		<LOQ	Mushroom, leaves, oil
9	415.1746	$C_{23}H_{28}O_7$	4.13, 4.26, 4.88, 5.25	249.0747, 221.0805, 207.0274		<LOQ	Mushroom, leaves, oil
10	425.1959	$C_{25}H_{30}O_6$	4.51. 4.63	207.0274		<LOQ	Mushroom, leaves, oil
11	431.1697	$C_{23}H_{28}O_8$	4.21, 4.42, 5.04	207.0274		<LOQ	Mushroom, leaves, oil
12	431.2062	$C_{24}H_{32}O_7$	4.85, 4.21, 4.41	207.0274		<LOQ	Mushroom, leaves
13	433.1854	$C_{23}H_{30}O_8$	4.08, 4.22, 4.35, 4.88, 5.17	207.0274		<LOQ	Mushroom, leaves, oil
14	451.2488	$C_{28}H_{36}O_5$	5.85, 6.08	207.0274, 282.0734, 249.0747	Eucalyptal D	<LOQ	Mushroom, leaves, oil
15	453.2633	$C_{28}H_{38}O_5$	5.86, 6.00, 6.12	249.0747, 207.0274	Eucalrobusones, eucalteretials, eucalypglobulusal, eucarobustols, euglobals, macrocarpals C, G	0.389	Mushroom, leaves
16	455.2427	$C_{27}H_{36}O_6$	4.88, 5.04, 5.24	207.0274, 249.0747		<LOQ	Mushroom, leaves
17	455.2790	$C_{28}H_{40}O_5$	4.16	249.0747		<LOQ	Mushroom
18	457.2942	$C_{28}H_{42}O_5$	4.00, 4.25, 4.51	207.0274, 249.0747		<LOQ	Mushroom, leaves
19	459.3099	$C_{28}H_{44}O_5$	4.43, 4.61	249.0747		<LOQ	Mushroom
20	467.2424	$C_{28}H_{36}O_6$	5.24, 5.08, 5.37, 4.83	249.0747, 207.0283, 282.0734	Eucalrobusones, eucalyptal A/C	0.0201	Mushroom, leaves, oil

FPC #	*m/z*	Molecular formula	t_R (min)	Product ion(s)	Putative ID(s)	Macrocarpal A equivalent (μg/g)	Detected in
21	469.2582	$C_{28}H_{38}O_6$	5.24	249.0747, 207.0274		0.0396	Mushroom, leaves, oil
22	471.1653	$C_{25}H_{28}O_9$	4.53, 4.45, 4.63, 4.92	221.0805, 249.0747, 282.0734	Loxophlebal A/B	<LOQ	Mushroom, leaves, oil
23	471.2736	$C_{28}H_{40}O_6$	4.72, 4.88, 5.17, 5.28	249.0747, 207.0274	Eucalyptins, macrocarpals A-F, H, K, L, M, N, O, Q, eucalteretial E, eucalypglobulusal G, eucarobustol C/F, euglobal IIIa, Euvimal-1, rhodomyrtal A/B	0.483	Mushroom, leaves, oil
24	473.1802	$C_{25}H_{30}O_9$	5.24, 5.11, 5.34, 6.12	249.0747, 155.0696, 181.0131, 223.0963	Robustaol A	<LOQ	Mushroom, leaves, oil
25	473.2889	$C_{28}H_{42}O_6$	5.19, 4.51, 4.22	249.0747, 181.0131, 223.0965		0.00630	Mushroom, leaves
26	481.2217	$C_{28}H_{34}O_7$	4.78, 4.88, 5.24, 5.64	207.0274, 249.0747, 282.0734		<LOQ	Mushroom, leaves, oil
27	483.2372	$C_{28}H_{36}O_7$	4.59, 4.78, 4.95, 5.24	249.0747, 207.0274, 233.0823		<LOQ	Mushroom, leaves, oil
28	485.1813	$C_{26}H_{30}O_9$	4.55, 4.47, 4.87	235.0963, 249.0747, 282.0738		<LOQ	Mushroom
29	485.2535	$C_{28}H_{38}O_7$	4.21, 4.45, 4.88, 5.35, 5.48, 6.12	235.0963, 249.0747, 207.0274	Eucalyptone, eucalypglobulusal A, eucalyptal B/E, macrocarpal N, P	0.358	Mushroom
30	487.2692	$C_{28}H_{40}O_7$	4.13, 4.33, 4.54	207.0274, 249.0747, 181.0131	Eucalypglobulusal B/E	0.0463	Mushroom
31	489.2842	$C_{28}H_{42}O_7$	3.99, 4.21	207.0274, 249.0747		<LOQ	Mushroom
32	499.1609	$C_{26}H_{28}O_{10}$	5.52, 5.27, 5.99	207.0274	Grandial, Jensenal, sideroxylonal A/B/C	3.17	Mushroom, leaves
33	499.2322	$C_{28}H_{36}O_8$	4.27, 4.40, 4.53, 5.15	249.0747, 181.0131 207.0274		<LOQ	Mushroom, leaves

FPC #	*m/z*	Molecular formula	t_R (min)	Product ion(s)	Putative ID(s)	Macrocarpal A equivalent (µg/g)	Detected in
34	503.2635	$C_{28}H_{40}O_8$	4.39, 4.08, 5.39	207.0274, 249.0747, 235.1332		0.000336	Mushroom, leaves, oil
35	515.1549	$C_{26}H_{28}O_{11}$	4.89, 5.92	249.0747, 207.0274		0.00761	Mushroom, leaves
36	519.2573	$C_{28}H_{40}O_9$	3.72, 4.24, 4.08	249.0747, 207.0274		<LOQ	Mushroom, leaves
37	551.2271	$C_{31}H_{36}O_9$	5.80	249.0747, 181.0131, 301.1433		<LOQ	Mushroom
38	607.2160	$C_{31}H_{36}O_{10}$	5.04, 5.18	357.1331, 249.0747, 181.0131		<LOQ	Mushroom
39	635.2842	$C_{33}H_{36}O_{11}$	5.04, 5.18, 4.70	249.0747, 181.0131, 385.2151		0.186	Mushroom, leaves
40	649.2632	$C_{36}H_{44}O_{10}$	5.42, 5.79, 5.26	399.1792, 249.0747, 181.0131		<LOQ	Mushroom, leaves

Table S3- Retention time and high resolution MS/MS fragmentation of commercially available eucalyptus standards

Compound	Molecular formula	*m/z* $[M-H]^-$	R_t, C8 (min)	Fragment ions*				
				F1	F2	F3	F4	F5
Euglobal Ia1	$C_{23}H_{30}O_5$	385.2020	4.87	249.0772	199.9550	343.9402	120.4508	125.8187
Euglobal Ia2	$C_{23}H_{30}O_5$	385.2020	4.91	249.0772	247.0609	207.0295	341.2120	189.8241
Macrocarpal C	$C_{28}H_{38}O_5$	453.2646	5.56	207.0294	282.0747	425.2708	193.0135	251.0927
Macrocarpal A	$C_{28}H_{40}O_6$	471.2752	4.33	207.0293	282.0746	443.2807	193.0135	251.0925
Macrocarpal D	$C_{28}H_{40}O_6$	471.2752	4.27	207.0288	282.0735	251.0921	193.0126	443.2802
Macrocarpal E	$C_{28}H_{40}O_6$	471.2752	4.28	207.0288	282.0738	251.0921	193.0136	443.2796
Macrocarpal N	$C_{28}H_{38}O_7$	485.2557	4.04	207.0296	251.0928	282.0750	457.2606	193.0137
Eucalyptone	$C_{28}H_{38}O_7$	485.2557	4.02	207.0298	249.0773	235.0970	282.0760	457.2609
Sideroxylonal A	$C_{26}H_{28}O_{10}$	499.1609	5.29	249.0771	181.0136	247.0616	305.1397	221.0820

*Fragment ions F1-F5 are listed in order of decreasing relative abundance in their MS/MS spectra, where F1 corresponds to the base peak

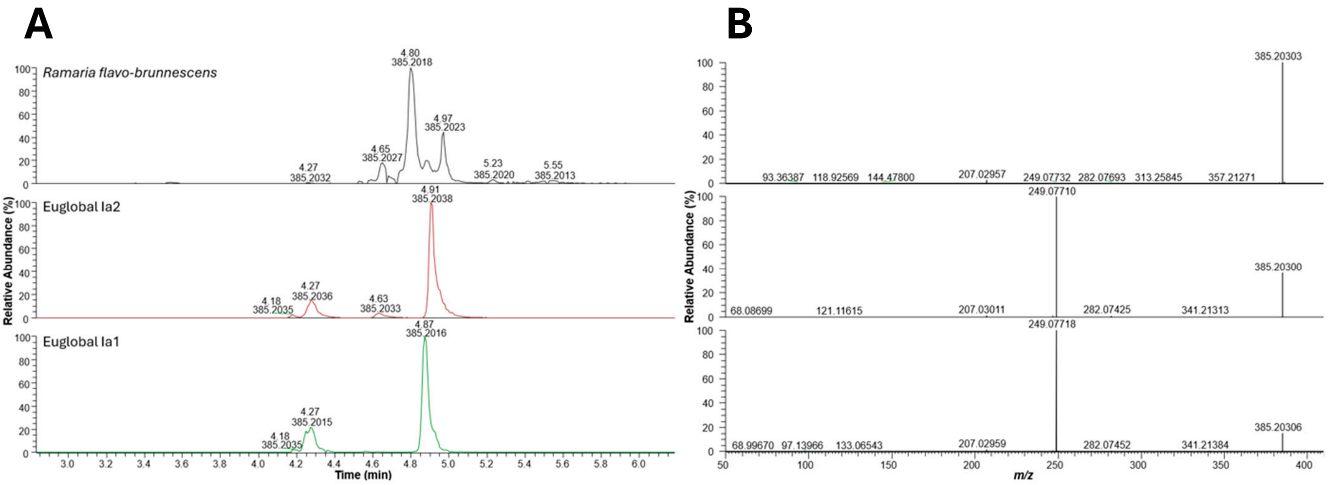

Figure S3- **A**) Extracted ion chromatograms of 385.2029 [M-H]- and **B**) MS/MS for the *Ramaria* extract, euglobal Ia1 and euglobal Ia2

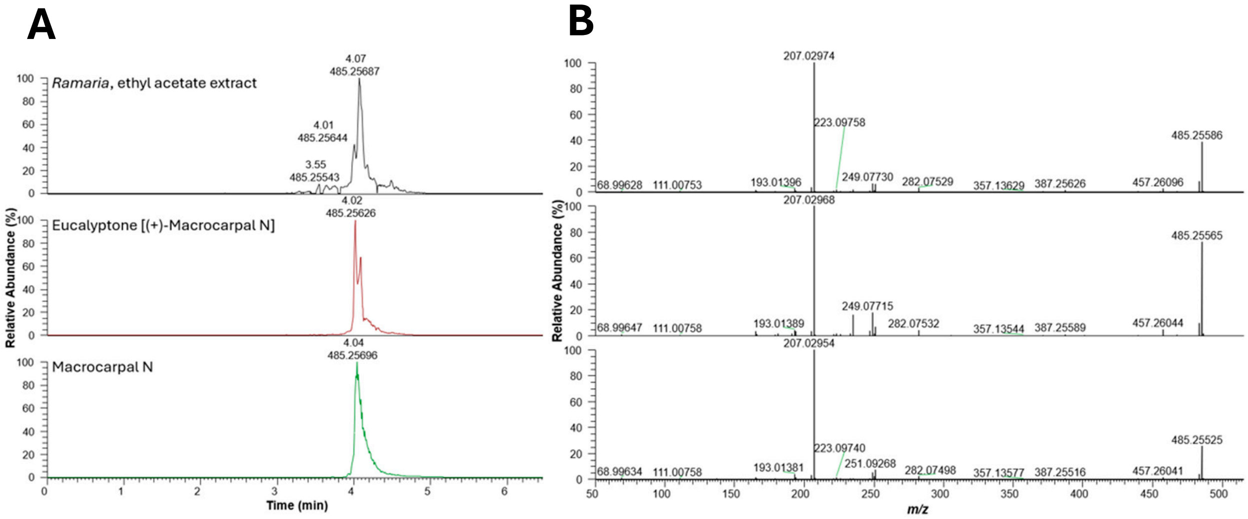

Figure S4- **A**) Extracted ion chromatogram of 485.2568 $[M-H]^-$ and **B**) MS/MS fragmentation for the *Ramaria* extract, eucalyptone, and macrocarpal N

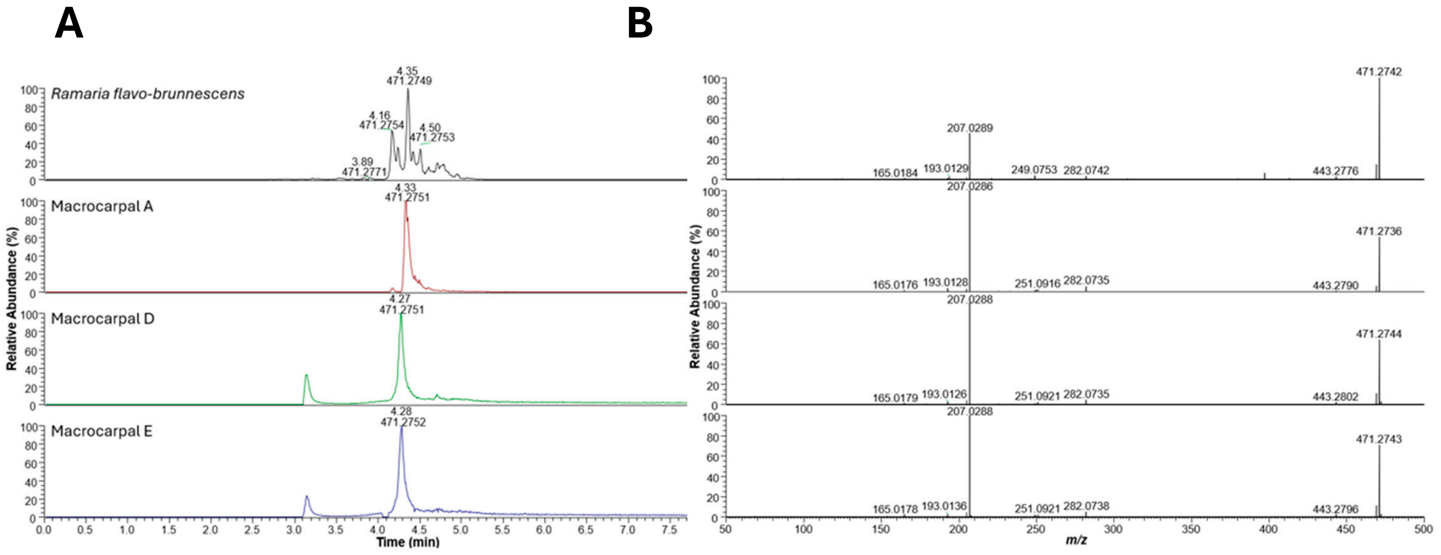

Figure S5- A) Extracted ion chromatogram of 471.2772 [M-H]$^-$ and **B**) MS/MS fragmentation of the *Ramaria* ethyl acetate extract, and macrocarpals A, D and E

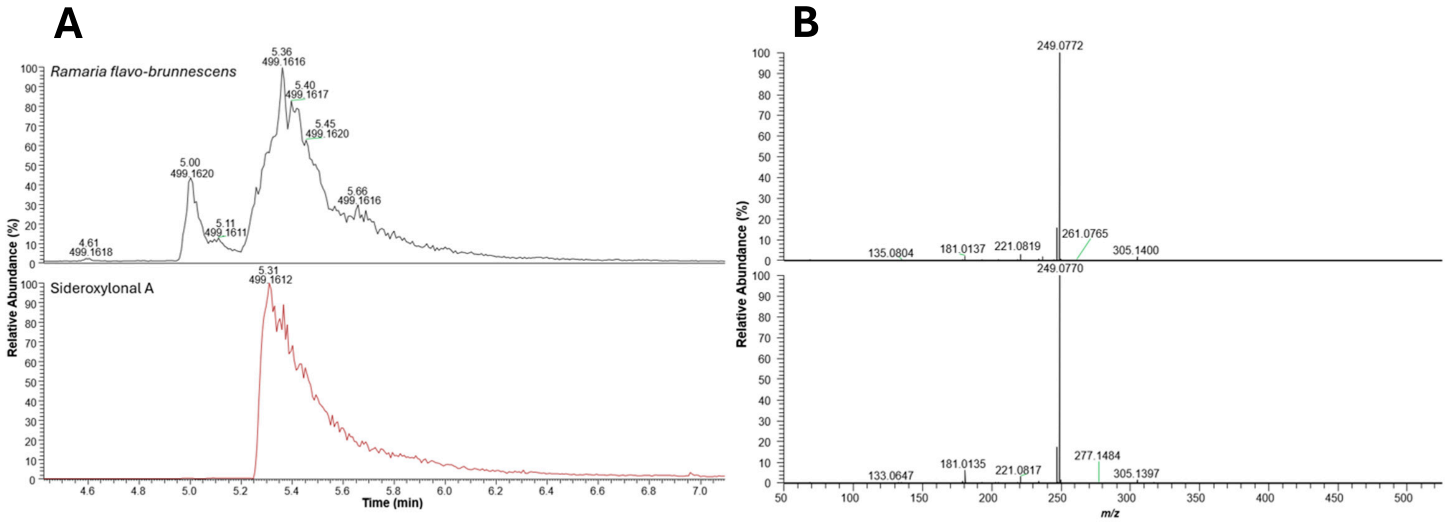

Figure S6-A) Extracted ion chromatogram of 499.1609 [M-H]$^-$ and **B**) MS/MS fragmentation of the *R. flavo-brunnescens* extract, and sideroxylonal A

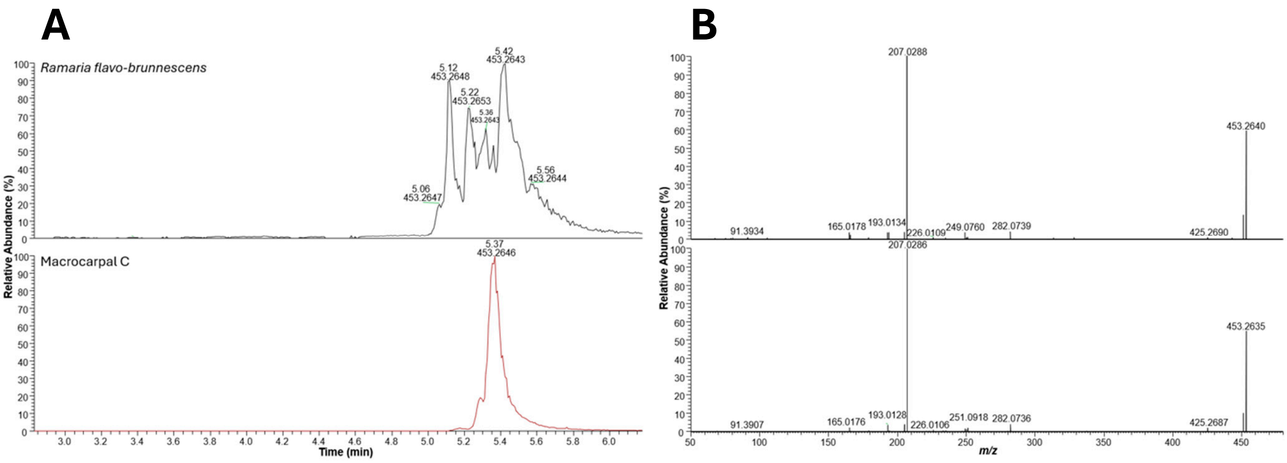

Figure S7- A) Extracted ion chromatogram of 453.2646 [M-H]$^-$ and B) MS/MS fragmentation of the *R. flavo-brunnescens* extract, and macrocarpal C
